# Drug use and nightlife: more than just dance music

**DOI:** 10.1186/1747-597X-6-18

**Published:** 2011-07-27

**Authors:** Tina Van Havere, Wouter Vanderplasschen, Jan Lammertyn, Eric Broekaert, Mark Bellis

**Affiliations:** 1 Department of Social Work and Welfare Studies, University College Ghent, Voskenslaan 362-370, 9000 Ghent, Brussels, Belgium; 2 Department of Orthopedagogics, Ghent University, H. Dunantlaan 2, 9000 Ghent, Belgium; 3 PC-lab of the Faculty of Psychology and Educational Sciences, Ghent University, H. Dunantlaan 1, 9000 Ghent, Belgium; 4 Department of Orthopedagogics, Ghent University, H. Dunantlaan 2, 9000 Ghent, Belgium; 5 Centre for Public Health, Liverpool John Moores University, 5th Floor Kingsway House, Hatton Garden, Liverpool, L3 2AJ, UK

## Abstract

**Background:**

Research over the last decade has focused almost exclusively on the association between electronic music and MDMA (3,4-Methylenedioxymethamphetamine or "ecstasy") or other stimulant drug use in clubs. Less attention has been given to other nightlife venues and music preferences, such as rock music or southern/funky music. This study aims to examine a broader spectrum of nightlife, beyond dance music. It looks at whether certain factors influence the frequency of illegal drug and alcohol use: the frequency of going to certain nightlife venues in the previous month (such as, pubs, clubs or goa parties); listening to rock music, dance music or southern and funky music; or sampling venues (such as, clubs, dance events or rock festivals). The question of how these nightlife variables influence the use of popular drugs like alcohol, MDMA, cannabis, cocaine and amphetamines is addressed.

**Methods:**

The study sample consisted of 775 visitors of dance events, clubs and rock festivals in Belgium. Study participants answered a survey on patterns of going out, music preferences and drug use. Odds ratios were used to determine whether the odds of being an illegal substance user are higher for certain nightlife-related variables. Furthermore, five separate ordinal regression analyses were used to investigate drug use in relation to music preference, venues visited during the last month and sampling venue.

**Results:**

Respondents who used illegal drugs were 2.5 times more likely to report that they prefer dance music. Goa party visitors were nearly 5 times more likely to use illegal drugs. For those who reported visiting clubs, the odds of using illegal drugs were nearly 2 times higher. Having gone to a pub in the last month was associated with both more frequent alcohol use and more frequent illegal substance use. People who reported liking rock music and attendees of rock festivals used drugs less frequently.

**Conclusions:**

It was concluded that a more extended recreational environment, beyond dance clubs, is associated with frequent drug use. This stresses the importance of targeted prevention in various recreational venues tailored to the specific needs of the setting and its visitors.

## Background

Epidemiological studies have shown that so-called party people (a global term for people who visit clubs, parties of all kinds, music festivals and dance events) are more experienced with illegal drugs than other groups of young people who "go out" [1-6]. A recent study by Calafat et al. [7] demonstrated that factors associated with various recreational nightlife activities, such as music preference and venue choice, were relevant predictors of illegal drug use in several European countries. Thus, studying the relationship between particular music preferences, or behavioural patterns of "going out" and illegal drug use, may help to identify potential pathways for targeted interventions to reduce drug-related harm among at-risk groups [8]. However, over the last decade, such research has focused almost exclusively on the correlation between electronic music and MDMA or other stimulant drug use [1,9,10]. While typical "club drugs", such as MDMA (XTC), cocaine (coke) and amphetamines (speed), have been closely linked to dance music [5,11-13], significantly less attention has been given to other music preferences, such as rock music.

This study aims to examine a broader spectrum of nightlife, beyond dance music. It looks at whether certain factors influence the frequency of illegal drug and alcohol use: the frequency of going to certain nightlife venues in the previous month (such as, pubs, clubs or goa parties); listening to rock music, dance music or southern and funky music; or sampling venues (such as, clubs, dance events or rock festivals).

Research on nightlife venues (e.g. festivals and pubs) other than clubs/raves is rare [14]. Calafat et al. [15] broadened the scope of their study to include other mainstream nightlife venues, demonstrating that the use of alcohol and illegal drugs is also linked to the frequency of visiting bars and pubs. Until recently, little attention has been paid to music festival attendees' use of illegal substances (these music festivals are comparable to rock festivals examined in our study). Lim et al. [8] interviewed young people attending a music festival in Australia, and found higher drug use prevalence among this population than among respondents to a National Drug Strategy Household Survey. In contrast, according to a UK survey, the majority of respondents reported that they did not use illegal drugs while attending music festivals [14]. Alcohol consumption was, however, reported by the majority (88%) [14]. Another study, which focused on first time use of legal and illegal drugs at music festivals, demonstrated that visitors to rock festivals mainly reported using tobacco and cannabis for the first time [16].

Moore and Miles [17] found an association between substance use and alternative music styles in the electronic music scene: respondents were more likely to consume MDMA at "hard house" and "trance nights", and were more likely to drink alcohol if they attended "funky house nights". According to key informants and police sources in Belgium, frequenters of goa parties are more likely to use drugs than people who frequent other sub scenes within the electronic dance music world [18]. Goa trance (common at goa parties) is essentially "dance-trance" music; the goal being to assist dancers to experience a collective state of bodily transcendence, similar to that of ancient shamanic dancing rituals, through hypnotic, pulsing melodies and rhythms. It has its roots in the state of Goa in India [19]. Although a shift in the dance party scene away from "underground" events has been observed [20], a revival of the dance "underground" has recently been reported, with the advent of goa parties held at secret venues [18]. Moreover, this alternative music style within the electronic dance music scene seems to be associated with greater drug use.

Belgium offers an excellent opportunity to focus on several different nightlife scenes, since it is known for its variety of music styles and venues and its large music events (such as, I Love Techno, 10 daysoff and rock festivals like Rock Werchter, etc.). This study will elaborate and expand upon findings from previous studies, because few studies have examined substance use by followers of various music styles (dance, rock and mixed southern and funky music) and frequenters of various nightlife venues (dance events, clubs, rock festivals, pubs, goa parties, etc.). These nightlife variables will be studied in relation to substance use. Although MDMA is the most notorious club drug [9,10,21-23], cannabis appears to be the most popular illicit drug among party people [11,24]. Furthermore, the combination of alcohol and illegal drugs or the combination of different illegal drugs, is a particularly worrying characteristic of dance drug users for policymakers and health workers [13,25]. This article focuses on the (frequency of) use of the most popular drugs: alcohol, cocaine, MDMA, cannabis and amphetamines [24].

## Method

### Study sample

This survey was administered to those participating in Belgian nightlife using a self-report questionnaire. A sample of 811 respondents was surveyed at three dance clubs, two dance events and two rock festivals in Flanders (for a more detailed description, see [24]). These specific events and clubs were chosen because of their scale (in order to ensure a large enough sample size) and location (regional spread). Furthermore, pragmatic issues played a role, like: already existing contacts with key figures in some regions and club owners, promoters, to maximize participation in the study. The most popular clubs and events in Belgium were included in the study. Dance music was played in the clubs and at the dance events, but there were also DJs or bands playing dance music at the rock festivals. The clubs included in this study are small scale, open every weekend, and have fewer visitors per occasion than the dance events or rock festivals, which are large events mostly held in the open air during the summer welcoming over 10 000 visitors.

In total, 1406 individuals were invited to participate in this study, 811 individuals completed the questionnaire, 595 people refused to participate. Based on the researchers' observations, many people refused to participate because they were on their way to a bar, or wanted to see a particular artist who was starting his/her act. Other people did not want to participate because they were accompanied by a group of friends. Surveys from 36 respondents (4.1%) were excluded from the data analyses as unreliable, because they reported the use of 'NTSC', an imaginary substance that was added to the questionnaire. Obviously intoxicated visitors were also barred from participating in this study. If these respondents insisted on participating, their filled in survey was marked for deletion and removed from the analyses (*n *= 3).

The final study sample of 775 respondents consisted of 61.9% males and 38.1% females, with a mean age of 22 years and 8 months (*M = 22.7, SD = 5.9*). The mean age of the sample recruited in clubs is lower as compared with the other two samples and more club visitors still live with their parents (F(2, 717 = 12.29, p < 0.01).). In the sample of respondents of dance events we find fewer respondents who are still students in comparison with rock festivals and clubs (χ² (2) = 30,37, p < 0.01). Furthermore, more female respondents were recruited at rock festivals (χ² (2) = 29,51, p < 0.01) (cf. Table 1).

**Table 1:** Sample characteristics (*n *= 775).

	*Dance events* *N (%)*	*Rock festivals* *N (%)*	*Clubs* *N (%)*	*Total* *N (%)*
**Respondents**	270 (34.8)	269 (34.7)	236 (30.5)	775

**Gender**				
**Male**	189 (25.3)	127 (17.0)	146 (19.6)	462 (69.1)
**Female**	74 (9.9)	131 (17.6)	79 (10.6)	284 (38.1)

Age				
**< 18**	26 (3.6)	74 (10.3)	54 (7.5)	154 (21.4)
**18-23**	117 (16.3)	86 (11.9)	107 (14.9)	310 (43.1)
**24-29**	79 (11.1)	54 (7.5)	40 (5.6)	173 (24.0)
**≥ 30**	26 (3.6)	47 (6.5)	10 (1.4)	83 (11.5)

**Occupation**				
**Student**	78 (10.8)	137 (19.1)	117 (16.3)	332 (46.2)
**Job**	166 (23.1)	113 (15.7)	108 (15.0)	387 (53.8)

**Living status**				
**With parents**	162 (21.4)	163 (21.5)	171 (22.6)	496 (65.5)
**On their own or living together with friend/partner**	99 (13.1)	101 (13.3)	61 (8.1)	261 (34.5)

### Procedure and survey instrument

This survey was first administered in 2003 and repeated in 2005 and 2007. In this paper, however, only the data from the survey conducted between 1 July 2007 and 12 November 2007 are included.

Visitors to dance clubs and events were asked to participate in this study. To avoid selection bias, polltakers invited every fifth visitor to complete a short self-report questionnaire. Visitors were informed that filling out the questionnaire would take 5 to 10 minutes; respondents received no financial compensation.

The questionnaire consisted of multiple-choice questions and two open-ended questions. The first section included some demographic variables [26], while the second part focused on going out patterns [5]. Eighteen music styles were listed in the questionnaire and study respondents cited their favourite music preference(s) (no limitations were imposed on possible answers). Categories for music preferences were based on an Internet search of relevant websites, and were approved during an expert meeting of prevention workers and nightlife professionals [27]. Another question asked respondents to select the nightlife venues they had visited in the last 30 days (e.g. pubs, clubs and goa parties). The third part of the survey instrument assessed the frequency of the use of various substances [5,13]. Seven categories for frequency of drug use were distinguished: 1) never used this drug; 2) ever used, but not in the last year; 3) once a month or less; 4) several times a month; 5) once a week; 6) several times a week; and 7) daily. The fourth section included questions on the context of substance use (e.g. when and with whom respondents use), as well as combined substance use or poly-drug use [28]. Every respondent was asked to complete the section on preventive health measures taken regarding substance use [29]. The last section of the questionnaire consisted of open-ended questions on emerging trends in (i.e. patterns of) drug use.

### Data analysis

Prior to the statistical data analysis, all questionnaires were first entered into the data set by hand, using SPSS 17.0. To ease the interpretation, some variables were recoded. To analyse the association between music preference and drug use, the 18 music styles included in this study were reduced to 3 music preferences based on media sources (e.g. Internet), interpretations of DJ's and observations from polltakers. In addition, we considered the internal consistency of the music categories. The above resulted in three categories of music preferences: dance music (*α *= 0.7), rock music (*α *= 0.6) and a mix of southern and funky music (*α *= 0.7) (cf. Table 2). "Dance music" included house, progressive, techno, electro, drum & bass, goa trance and trance. "Rock music" consisted of rock, surf, metal, hardcore and pop music. "Southern and funky music" included Salsa, Latino and R&B, hip-hop and rap, disco, reggae and ragga.

**Table 2:** Proportion and odds of last year substance use according to music preference, nightlife environment and sampling venue (*n *= 775).

	% that used any illegal drug during the last year	OR	95% CI
Music preference (yes/no)			

Dance music	55.6	2.47**	1.61 - 3.78

Rock music	46.1	0.53**	0.39 - 0.72

Southern and funky music	53.9	1.16	0.86 - 1.56

Visits to (yes/no)			

Clubs	57.9	1.79**	1.33 - 2.42

Pubs	51.5	0.99	0.66 - 1.48

Goa parties	82.5	4.85**	2.41 - 9.77

Sampling venue (yes/no)			

Dance events	56.5	1.34	0.99 - 1.82

Rock festivals	41.9	0.54**	0.40 - 0.74

Clubs	57.8	1.42*	1.03 - 1.94

Fisher Exact Probability Tests with * *p *< 0.05, ** *p *< 0.01 and *** *p *< 0.001.

Given the variety of substances included in the questionnaire, we limited our findings to the substances most frequently used by those participating in Belgian nightlife: alcohol, cannabis, MDMA, amphetamines and cocaine [24].

To determine the relation between substance use and nightlife variables, two types of analyses were performed. First, to determine whether the odds of being an illegal substance user are higher for certain music and nightlife-related variables (i.e. music preference: rock, dance and southern/funky music; last month visits: clubs, pubs and goa parties; sampling venues: dance events, rock festivals and clubs), we calculated odds ratios for the subsample of respondents who claimed to have used an illegal drug during the last year and the subsample of those who did not. Focusing on use last year gives a more reliable insight than focusing on more recent use, because the latter category could be influenced by the timing of the survey: during holidays and free of responsibilities, young people tend to use more substances than during the school year [30]. We compared last year illegal drug use (yes/no) with the nightlife related variables (yes/no variables for dance, rock and southern/funky music, going to pubs, clubs, goa parties, sampled at dance events, rock festivals or in clubs). Second, to investigate the association between the frequency of use for specific types of drugs and the various independent variables, we performed five separate ordinal regression analyses using a proportional odds model [cf. [31]]. In each of these five analyses, the frequency of using a specific substance during the last year (alcohol, cannabis, amphetamines, MDMA or cocaine) was regressed on age (entered as continuous variable), gender, music preference (yes/no for dance music preference, southern and funky music preference, rock music preference), number of visits to clubs, pubs and goa parties within the last month (entered as continuous variables), and sampling venue. To interpret venue effects with regard to the grand mean, the original venue variable consisting of three categories was recoded using an effect coding scheme [32].

For practical reasons (because more categories increase the difficulty of data interpretation from ordinal regression analyses), the original dependent variable (frequency of last year use) consisting of 7 categories was reduced into a variable with three ordered categories: (1) No use: people who never used this drug or have used it, but not within the last year; (2) Occasional use: people who recently used this drug, on a monthly basis or less frequently; and (3) Regular use: people who used this substance at least weekly. As opposed to simple logistic regression, using these ordered categories enables us to investigate the frequency of last year use instead of simply having used a certain drug the last year (yes/no). In the parameterization used by Stata for the proportional odds model, a positive value for β indicates that with increasing values for the predictor, the odds increase of being above a given value of k (with k = 1, ..., number of ordinal categories - 1). In other words, a positive coefficient implies increasing probability of being in higher-numbered categories (of the dependent variable Y) with increasing values for the predictor (holding all other independent variables fixed) [32]. For the present analyses this means that a positive coefficient points to an increased probability of being a high frequency user.

The proportional odds assumption was not fulfilled for models with alcohol, cannabis and MDMA use as the dependent variable. However, fitting partial proportional odds models for these cases did not alter data interpretation. All (partial) proportional odds model fitting was done using STATA 10 [33,34]. The statistical significance level was set at *α *= 0.05.

## Results

### Going out patterns

A total of 775 visitors to clubs (30.5%), dance events (34.8%), and rock festivals (34.7%) were included in the study. Over four-fifths (84.6%) of the survey respondents reported that they liked dance music, 61% liked rock music, and more than half of the respondents liked southern and funky music (53.9%). Based on our results, there is a clear overlap between the categories, suggesting that, nowadays, young people prefer various kinds of music.

When asked how many times they visited a nightlife venue in the last month, 84.4% of survey respondents reported having been in a pub and 57.1% reported visiting a club, while only 7.9% of respondents attended goa parties. The mean frequency of going to these venues in the last month is 7 times for pubs and 2 times for clubs.

### Drug use characteristics

Alcohol was the most commonly used party drug (91.5%) during the last year, while more than half of the respondents (51.8%) reported using an illegal drug. Cannabis (44.4%) was the most popular illegal substance, followed by MDMA (19.1%) and cocaine (17.1%), while the use of amphetamines was reported to a limited extent (10.0%). With respect to regular substance use, 63.9% of respondents consumed alcohol on a daily to weekly basis and 22.4% smoked cannabis with the same frequency. As expected, stimulants were used less frequently: 5.9% reported regular MDMA use, 4.3% regular cocaine use, and 3.5% regular amphetamine use.

### Illegal drug use over the last year

Odds ratios were calculated (cf. Table 2) in order to further examine the differences between the respondents who reported having used illegal drugs during the last year and those who reported not having used illegal drugs during the last year. The odds of respondents who reported liking dance music using illegal drugs were 2.5 times higher (*OR *= 2.47, 95% *CI *[1.61, 3.78]) than the odds of respondents not liking dance music using illegal drugs. Also, the odds of respondents that indicated liking rock music using an illegal drug were half as high as respondents who did not report liking rock music (*OR *= 0.53, 95% *CI *[0.39, 0.72]). Eight out of 10 goa partygoers reported using an illegal drug during the past year. In terms of odds, visitors of goa parties were nearly five times more likely to have used an illegal drug than respondents not going to these parties (*OR *= 4.85, 95% *CI *[2.41, 9.77]). Furthermore, the odds of respondents who reported frequenting clubs having used an illegal drug were almost twice as high as people not going to clubs (*OR *= 1.79, 95% *CI *[1.33, 2.42]). The proportion of illegal drug users was lower in the group of respondents who were recruited at rock festivals (*OR *= 0.54, 95% *CI *[0.40, 0.74]), but significantly higher for respondents recruited in clubs (*OR *= 1.42, 95% *CI *[1.03, 1.94]).

### Predictors of drug use related to music preference, last month visits and sampling venue

All music and nightlife-related variables, as well as gender and age, were entered into an ordinal regression analysis. The three-level (no, occasional and regular use) variables of last year use of alcohol, cannabis, MDMA, amphetamine and cocaine use were included as dependent variables to investigate the frequency of use. All of the models were significant (an overview of all related statistics is presented in Table 3).

**Table 3:** The proportional odds ratios and their 95% CI for the five final ordinal regression models.

	Alcohol		Cannabis		MDMA		Amphetamines		Cocaine	
	**OR**	**95% CI**	**OR**	**95% CI**	**OR**	**95% CI**	**OR**	**95% CI**	**OR**	**95% CI**

**Final model**										

**Age in years**	1.04**	1.01-1.08			1.10***	1.06-1.15	1.06*	1.01-1.11	1.09***	1.05-1.14

**Gender male**	0.55**	0.38-0.78	0.52***	0.37-0.73	0.55*	0.33-0.91			0.55*	0.32-0.94

**Music preference**										

**Dance music**			2.25**	1.36-3.72	3.19*	1.12-9.09			3.46*	1.08-11.07

**Southern/funky music**										

**Rock music**									0.47**	0.29-0.76

**Number of Last month visits**										

**Pubs**	1.08***	1.05-1.11	1.02*	1.00-1.04	1.05**	1.02-1.08			1.04**	1.01-1.07

**Clubs**	0.95*	0.90-1.00			1.11***	1.05-1.17			1.11***	1.05-1.18

**Goa parties**			1.28**	1.09-0.151			1.10*	1.02-1.19	1.23**	1.07-1.42

**Sampling venue**										

**Rock festivals**					0.41	0.26-0.65	0.50*	0.29-0.87	0.51**	0.31-0.81

**Dance events**					1.63**	1.19-2.23			1.69**	1.22-2.35

**Clubs**					1.48*	1.04-2.11				

**LR chi2 (10)**	62.28		70.57		109.25		37.59		124.45	

**Prob > chi2**	0.0001		0.0001		0.0001		0.0001		0.0001	

**Pseudo R squared**	0.058		0.054		0.145		0.078		0.180	

For each drug, last year use (no, occasional and regular use) was regressed on various socio-demographic, music and nightlife variables.

Wald z-tests with * *p *< 0.05, ** *p *< 0.01 and *** *p *< 0.001.

#### Age & gender

Age was found to be positively associated with higher levels of alcohol, MDMA, cocaine and amphetamine use. More specifically, when age increases, the odds of falling into higher categories (i.e. more frequent use) of alcohol, MDMA, cocaine and amphetamines use also increase (alcohol *OR *= 1.04, 95% *CI *[1.01, 1.08]; MDMA *OR *= 1.10, 95% *CI *[1.06, 1.15]; cocaine *OR *= 1.09, 95% *CI *[1.05, 1.14]; amphetamines *OR *= 1.06, 95% *CI *[1.01, 1.11]), but not for cannabis.

In addition, being male (gender) was identified as a factor that makes a significant contribution to higher use of all substances, except amphetamines. The odds of female participants falling into high categories of alcohol, cannabis, MDMA or cocaine use are approximately 50% lower than for male participants (alcohol *OR *= 0.55, 95% *CI *[0.38, 0.78]; cannabis *OR *= 0.52, 95% *CI *[0.37, .73]; MDMA *OR *= 0.55, 95% *CI *[0.33-0.91]; cocaine *OR *= 0.55, 95% *CI *[0.32, 0.94]).

#### Music preference

Dance music preference is positively related to the use of cannabis, MDMA and cocaine. More specifically, respondents who reported liking dance music have significantly higher odds of using cannabis, MDMA and cocaine more often than those that reported not liking dance music (cannabis *OR *= 2.25, 95% *CI *[1.36, 3.72]; MDMA *OR *= 3.19, 95% *CI *[1.12, 9.09], cocaine *OR *= 3.46, 95% *CI *[1.08, 11.07]).

Interestingly, liking rock music was found to be inversely associated with cocaine use. In fact, the odds that a rock music fan would fall into a higher category of cocaine use were a factor of 0.47 (95% *CI *[0.29, 0.76]) times smaller than respondents who said that they do not like rock music.

No significant associations were found between the preference for southern/funky music and the last year use of any of the drugs investigated.

#### Visits during the last month

With the exception of amphetamines, a positive association was found between the reported frequency of visiting a pub during the last month and higher use of all substances investigated; the more frequently respondents visited pubs, the higher the odds become that they fall into higher categories (more frequent use) for using alcohol (*OR *= 1.08, 95% *CI *[1.05, 1.11]), cannabis (OR = 1.02, 95% *CI *[1.00, 1.04]), MDMA (*OR *= 1.05, 95% *CI *[1.02, 1.08]) and cocaine (*OR *= 1.04, 95% *CI *[1.01, 1.07]).

The reported frequency of attending goa parties was found to be a predictive variable in the models for cannabis, amphetamine and cocaine use. More specifically, the more frequently respondents attended these parties during the month prior to the survey, the higher the odds of using cannabis (*OR *= 1.28, 95% *CI *[1.09, 1.51]), amphetamines (*OR *= 1.10, 95% *CI *[1.02, 1.19]) and cocaine (*OR *= 1.23, 95% *CI *[1.07, 1.42]).

Frequent club visits were positively associated with the use of MDMA and cocaine, but negatively associated with alcohol consumption. That is to say, the greater the frequency of club visits the greater the odds of higher levels of MDMA (*OR *= 1.11, 95% *CI *[1.05, 1.17]) and cocaine (*OR *= 1.11, 95% *CI *[1.05, 1.18]) use. But at the same time, the greater the frequency of club visits was the lower the odds of high levels of alcohol consumption were (*OR *= 0.95, 95% *CI *[0.90, 1.00]).

## Sampling venue

Compared to the average substance use level during the last year, respondents recruited at rock festivals reported less frequent use of MDMA (*OR *= 0.41, 05% *CI *[0.26, 0.65]), amphetamines (OR = 0.50, 95% *CI *[0.29, 0.87]) and cocaine (OR = 0.51, 95% *CI *[0.31, 0.81]). Whereas, compared to average levels of reported MDMA usage, participants recruited at clubs reported higher levels (*OR *= 1.48, 95% *CI *[1.04, 2.11]).

For participants recruited at dance events, the reported levels of both MDMA and cocaine use were significantly higher than average (MDMA *OR *= 1.63, 95% *CI *[1.19, 2.23]; cocaine *OR *= 1.69, 95% *CI *[1.22, 2.35]).

## Discussion

This study aims to examine a broader spectrum of nightlife, beyond dance music. Therefore, we questioned 775 visitors of clubs, dance events and rock festivals on their patterns of going out and drug use.

### Dance music, dance events and clubs

Although legal and illegal substances were reportedly used at all nightlife venues and were associated with all music preferences, we uncovered some clear trends. As we expected the use of illegal drugs was more common at dance music venues than other nightlife venues. From our results, it is clear that illegal drug users (defined as those who have used an illegal drug during the past year) are more likely to report that they like dance music and frequently "go clubbing". More specifically, individuals who report liking dance music are more likely to use MDMA and cocaine frequently, two typical "club drugs", as well as cannabis. Respondents recruited at dance events reported higher MDMA and cocaine use and those recruited at clubs higher MDMA use.

Furthermore, frequent club visits (number of visits during the last month) were positively associated with the use of MDMA and cocaine. However, the frequency of club visits during the last month was negatively associated with alcohol consumption. Although not drinking alcohol was identified as a preventive measure by clubbers some years ago, we thought this trend had disappeared [18]. It is possible that some of the clubbers who use drugs are careful not to mix illegal drugs and alcohol in order to prevent health problems. Another reason clubbers do not drink alcohol might be that they believe that consuming alcohol leads to aggressive and violent behaviour [11]. Furthermore, party people themselves say alcohol does not taste very nice when taken with ecstasy or amphetamines. In addition, the effects of consuming alcohol are oppressed if it is combined with cocaine (from the perception of the user) but still followed by a hangover afterwards [35].

### Goa parties

Even though the notion of distinct subcultures may be outdated [36], there are still some sub scenes in which drug use plays a more prominent role, and in which a greater proportion of attendees use illegal drugs. In this study, participants who reported attending goa parties within the last month appeared to have had even more experiences with drugs than those who reported liking dance music. In fact, 8 out of 10 goa partygoers had used an illegal drug during the last year and were five times more likely to be illegal drug users. According to a study in 2005 [18], the odds of goa party visitors using illegal drugs were 20 times higher than the odds of non-goa party visitors using illegal drugs. More specifically, going to goa parties was found to be a predictor of more frequent use of cannabis, amphetamines and cocaine. Specific music is being played at goa parties: goa trance and psy trance. It seems that underground or elitist music preferences can be linked to a higher prevalence of illegal drug use [cf. [37]]. Mulder et al. [38] have also found that a preference for non-mainstream music was positively associated with substance use, while in a study by Measham and Moore use of drugs during the previous month was highest among hard dance visitors [39]. No relation was found with alcohol use, although some authors have stated that the combination of alcohol and illegal drugs is very common in this scene. Usually only light beers are sold at these parties [35].

### A more expanded nightlife environment

The use of substances was not linked exclusively with the reported frequency of visiting clubs or preference for dance music, but it was associated with a more expanded nightlife environment. In addition to more frequent alcohol use, going to pubs in the last month was associated with more frequent use of illegal substances, including cannabis, MDMA and cocaine. In support of this, Calafat et al. [15] found that, in addition to discos and after-hours venues, legal and illegal substance use was also linked (to an even greater extent) with nightlife recreational venues that are less significant in the techno culture, such as bars and pubs.

### The influence of rock music

Interestingly, reported preferences for rock music and being recruited at a rock festival, in contrast with other music styles or venues, appeared to be protective factors against substance use. Our results show that people who say they like rock music use cocaine less frequently than people who say they do to not like rock music. In addition, compared to the average, attendees of rock festivals were less likely to use illegal drugs such as MDMA, amphetamines and cocaine, although no clear association with alcohol use was observed. Furthermore, respondents recruited at rock festivals use cannabis less frequently than visitors at clubs and dance events [40]. In a sample of respondents recruited at music festivals in the UK [14], the majority (68%) did not use any illegal drugs, and the most commonly used drug was cannabis. Hesse concluded in Denmark that onset use of cannabis at festivals is common, but trying other illicit drugs for the first time was rare [16].

It remains unclear whether taking drugs makes an individual more likely to listen to certain types of music or whether preferring certain types of music makes an individual more likely to use drugs [1,8]. Party people use illicit drugs in recreational settings to enhance their musical and other social experiences [8]. Measham and Moore found evidence for a complex relationship between drug use, drinking, venue type and the entertainment on offer [39]. Respondents in a study by Moore & Miles [17] reported differences in the types of substances consumed, depending primarily on musical preference and venue. Thus, it appears more likely that adolescent substance users identify themselves according to their choice of music, while listening to music seems less likely to encourage drug use among this age group. In particular, more outgoing young people, who are able to socialise in the young adult world, would be most likely to discover rave music and drugs [1]. The problem is not, therefore, that young people fall victim to bad influences because they have other options. It is, rather, a matter of a two-dimensional dialectic: individual identity development as opposed to the prevailing culture [41,42]. Peers' drug use or the perception that drug use is one of the prerequisites for acceptance and integration [41,43] influences young party people newly arrived on the nightlife scene. Malbon [44] talks about 'belonging', feeling part of a group, and taking on the habits and patterns of a group. Peer groups provide their members with an identity and sense of belonging different to those expected by the family [45]. Mulder et al. [43] conclude that fans of different types of music select friends with use patterns that reinforce their own substance use inclinations.

### Prevention in the nightlife environment

Festivals, dance events and clubs offer an excellent opportunity for health promotion, as it is possible to reach a large number of at-risk people in a short period of time [8]. Rave-based harm reduction strategies are appropriate for reducing the potential negative consequences of drug use [10]. Environmental strategies in clubs are also believed to have the potential to develop effective drug prevention strategies [46]. However, based on our results, prevention efforts should not be limited to dance parties and clubs, because a considerable number of young people encounter drugs in other nightlife venues. Furthermore, as the results of the ordinal regression show we should make a distinction between occasional users (e.g. once a year) and more regular users.

Substance use prevention at rock festivals should focus on the use of alcohol and cannabis. Based on our results, going to rock festivals or listening to rock music does not appear to be directly linked to the use of other illegal drugs. However, prevention measures are still needed, including free water, especially when it is very hot outside. Rock festivals are mostly held in the summer and can attract tens of thousands of visitors (sometimes up to 80,000 people). The use of alcohol and/or cannabis in combination with the summer heat and other circumstances might cause health problems. Furthermore, music festivals may serve as an occasion for trying cannabis for the first time, and are therefore important targets for the prevention of cannabis use onset [16].

With the cooperation of pub owners, bartenders and bathroom personnel, very structured prevention and safety measures (e.g. staff training programmes) can be used to reach pub visitors. Such targeted interventions for reducing alcohol-related harm have been positively evaluated [47-50], and could be extended to illegal drug users. Also, providing health education materials at nightlife venues may be more effective than spreading anti-drug use messages. Whittingham et al. [51] found that exposing young people to health education materials about how to minimise the potential hazards associated with drug use, rather than solely discouraging the use of drugs, did not have any counterproductive effects on individual acceptance of party drug use or other risk behaviours.

A specific at-risk population are those who visit goa parties. As these parties are increasingly organised within the public scene, collaborations with party organisers should be developed in order to set up prevention activities. Organisers are generally willing to take some precautions, especially since most of them have experienced first-hand the potential health risks associated with visitors who misuse drugs [40]. Substance use prevention for this group of party people needs to be tailored to their specific situation, and should start from the premise that drug use is deeply-rooted within this scene. Like prevention programmes that target overheating and drunk/drug-influenced driving, the overall objective should be to minimise harm. Sumnall et al. [52] suggest that hedonistic young people should be targeted with messages that increasing healthy choices will lead to more years in which to experience happiness and fun. Possible prevention and safety measures could include (as at other dance music events or clubs): 1) providing free water; 2) setting up an information stand on drug use; and 3) setting up a "chill-out" area where people can go when they have a bad trip. In addition, improved training for medical staff could enable visitors suffering from drug intoxication to be appropriately assessed [53]. Goa parties typically last a whole weekend; music is played both at night and during the day. Designating peers to check whether people who appear to be sleeping are sleeping and not unconscious could therefore be of importance at these parties. Peer support interventions can be valuable. A recent evaluation of a peer-led intervention in Australia has suggested that peers are seen as credible sources of information and that messages delivered are remembered up to three months later [54].

Bringing your own food and drink is allowed at most goa parties. This is also an important preventive measure, because financial reasons might hold back people to drink non-alcohol drinks and eat regularly. Furthermore, whether substance use and the behaviour associated with it, such as meeting sexual partners, leads to increased well being or ill health depends on the environment and individuals' specific behaviour [55]. For example, the decorations at parties can stimulate the psychedelic experience which most goa party people are looking for, but the decorations can also introduce individuals to bad trips (e.g. chaotic decoration). Clear messages about associations with physical and psychological problems are needed for this group. Prevention efforts should account for contextual and motivational factors -- particularly the issues of pleasant and unpleasant times in the lives of young adults -- in order to reduce its associated adverse outcomes [56]. Prevention should differentiate between party people who experiment with drugs occasionally and party people who use drugs regularly (cf. results from the ordinal regression). More frequent users might reject messages on harm reduction. How pleasure can be incorporated into harm reduction should be central to the future development of policy and practice [57]. Those on the goa scene are, like hippies, against formal institutional structures and authority. Over-regulation could have stimulated young people to look to illegal parties for their entertainment. Initiatives taken in this scene will be better if they involve the goa party visitors themselves [55].

### Study limitations

First, although clubbers and visitors of music festivals and events are often difficult to reach for research surveys, we were able to recruit nearly 800 respondents from festivals, dance events and clubs. Although the polltakers cannot guarantee that respondents were not under the influence of a substance when they filled out the survey, intoxicated individuals were not allowed to participate. Several authors have shown that, even in party environments, questionnaires can be used as reliable tools for assessment [9,58]. By adding an imaginary substance to the list of substances, we controlled the reliability of respondents' answers and excluded unreliable surveys from the analyses.

A sample of visitors was selected from various nightlife venues. Although this study was not representative of all party people in Belgium, the most famous clubs and events in Belgium were included in this study. Belgium is well-known for its large music festivals, the variety of its music scene, and as the home of several pioneers of electronic music.

The chance of recruiting the same attendee at multiple events is very small and visitors would probably have pointed it out if they had already been asked to fill out the questionnaire. A more appropriate method for studying party people would be conducting a survey for an entire year at various venues. In addition, the use of online surveys would allow researchers to reach more respondents, although this could raise other methodological issues, like the problem of double counting or the representativeness of the study sample.

Second, the inclusion of only 18 different music preferences may have meant some participants had an inadequate number of options. Although respondents could fill in other music preferences, only a limited number of participants did so. In addition, answers to the question concerning which nightlife venues they had visited during the last 30 days could be dependent on the timing of the survey; sometimes the last 30 days included holidays or a period of school examinations. Dividing respondents into groups based on their music preferences and/or venue choices could also be questioned, because young people do not have only one music or venue preference [45]. However, we countered this problem by including and analysing frequencies instead of nominal values: more frequent participation in a nightlife scene was linked to the frequency of substance use.

Third, some questions were excluded after piloting the questionnaire. More interesting information could be included if the questionnaire could have been longer.

Finally, the frequency of substance use was divided into three categories, and failed to provide insight about the quantity of substances being used. Further research could yield more information on this topic.

## Conclusions

Dance music lovers and visitors of goa parties and clubs are more likely to use illegal substances than those who do not like these music and go to these venues. Further research in the goa scene is needed to explore why the frequency of substance use is high in this scene. Research on the relationship between drug use and music has focused almost exclusively on electronic music and MDMA or other stimulant drugs. However, our results indicate that the frequency of drug use is linked to a more extended recreational nightlife environment. Respondents who frequently visit pubs are more likely to have used alcohol and illegal drugs frequently. While, respondents recruited at rock festivals are the least likely to have used an illegal drug in the past year, and respondents who like listening to rock music used cocaine less frequently. However, young people who go out cannot be simply classified in one category. Dance music lovers also go to rock festivals or pubs, while rock music lovers will sometimes visit parties in clubs. This stresses the importance of prevention activities that target various recreational nightlife venues, and are tailored to the specific needs of the setting and its visitors.

## Competing interests

The authors declare that they have no competing interests.

## Authors' contributions

THV carried out the survey, helped with the statistical analysis and drafted and revised the manuscript. WVP helped to draft and revise the manuscript. JAL performed the statistical analysis and revised the manuscript. EBR and MAB gave feedback on the manuscript and statistical analysis. All authors read and approved the final manuscript.
